# The Committee Stage of the National Insurance Bill

**Published:** 1911-07-22

**Authors:** 


### THE COMMITTEE STAGE OF THE NATIONAL INSURANCE BILL.

The Committee is plodding slowly through the clauses of the National Insurance Bill, but up to the present only nine clauses have been considered. The House will shortly be engaged with the Parliament Bill, and as there are other measures of first-rate importance which it is intended to pass this session, it is quite probable that before long the Government may deem it expedient to make a time-limit for the Insurance Bill. Clause 8, the consideration of which began last week, occupied several sittings, and gave rise to an interesting discussion on the value of the sanatorium treatment of tuberculosis. The debate arose on an amendment moved by Mr. Austin Chamberlain to omit the second paragraph of the clause, which provides that the benefits conferred on insured persons shall include treatment for tuberculosis in sanatoria. He argued that such treatment did not properly come within the province of the Bill, and, further, that for the present it would be better to devote more money to research work on tuberculosis and cancer. Mr. Long expressed the opinion that to erect a chain of sanatoria throughout the kingdom would be premature, as our knowledge as to the real effect of the treatment was limited. Dr. Addison supported the policy of building sanatoria with the reservation that too much should not be spent on "bricks and mortar." He mentioned that at the present time only two thousand beds were available in sanatoria for the whole community, of which only fifteen hundred could be obtained by people with small means; on the other hand, there were over a quarter of a million persons in the country suffering from tuberculosis. The Ancient Order of Foresters pay on an average in 4,707,608 cases of sickness annually, and of these 941,575, or ·20 per cent., is due directly to phthisis, and at least 51 per cent. to other forms of tuberculosis. The Hearts of Oak estimate that the average cost of each consumptive invalid is £41, while for other invalids it is £23 each. Friendly societies at the present time spend more than a million a year in treating this disease. Were not these facts, asked Dr. Addison, sufficient to justify the inclusion of provisions for sanatoria in a national scheme of insurance? He agreed that it was necessary to have some provision in the Bill to follow up cases after they had left the sanatorium. Dr. Hillier could not defend the policy laid down in the Bill, which was not that of treating the disease as a whole, but merely offering a sort of palliative and making a sort of beginning. The greatest source of the spread of the disease was to be found in those advanced cases of tuberculosis which are allowed to go home to die or to remain there in a narrow little home or in a crowded tenement. Mr. Henry Norman and Mr. Arthur Lynch having contributed to the debate, Mr. Barnes expressed approval of the provisions for sanatoria, and said he should vote every time he got a chance for extending the principle so as to make it theoretically more perfect. The Chancellor of the Exchequer, in replying to the various criticisms, assured the House that there was no intention of relying exclusively on sanatorium treatment; it would be supplemented by other methods, for it was not every consumptive patient who could be sent profitably to a sanatorium. With regard to the demand for the inclusion of women and children, he said the sanatorium benefit might be extended to the families of insured persons with the co-operation of the local authorities, for the Bill provided that the State might make good half of the excess expenditure of Local Health Committees for medical benefit, the local authorities making good the other half; this arrangement might be made to apply in the case of sanatorium benefit also. Among the subsequent speakers were Sir Randolph Baker, who suggested that instead of building sanatoria something might be done by using tents; Dr. Chapple, who thought it would be generally accepted that better results in the cure of tuberculosis had followed the erection of sanatoria than any other treatment; and Dr. Esmonde, who trusted the Chancellor would see if something could be done to continue the open-air treatment at the houses of the people themselves. Mr. Chamberlain's amendment was negatived, and an amendment, moved by Mr. Lansbury, to extend the sanatorium benefit to the wives and children of insured persons, was withdrawn, after the Chancellor had made the statement referred to above.

In the course of Monday's discussion, the Chancellor announced an important concession with regard to maternity benefit. An amendment having been moved with the object of granting medical benefit to persons entitled to maternity benefit, the Chancellor refused to go so far, but moved an amendment, which was agreed to, providing that a wife who worked and was insured should receive sick pay of 7s. 6d. a week in addition to maternity benefit. Clause 9, which provides for reduced rates on benefit in certain cases, was passed, with some modification, on Tuesday night.

When the clauses mainly affecting the position of hospitals under the proposed scheme are reached, a number of amendments, of some of which notice has been given, will be moved. The amendment of Clause 15, desired by the General Medical Council, provides that voluntary hospitals should be dealt with in the same manner as sanatoria and other similar institutions. With regard to Clause 17, the Council thinks that if voluntary hospitals are constrained, for financial reasons, to accept the conditions laid down by friendly societies, subject to no review by the Insurance Commissioners or the Advisory Committee, their efficiency may be impaired; voluntary hospitals should be omitted from this clause. The Council suggests that the Local Health Committee should be empowered to make arrangements, to the satisfaction of the Insurance Commissioners, with hospitals for the treatment of insured persons therein, and that no well-conducted hospital objects to visitation and inspection by competent persons. At the time of writing the amendment embodying the principle of the resolution adopted at Monday's meeting of members and representatives of charitable societies had not appeared on the paper. An amendment of Clause 17, of which notice has been given by Mr. Archer-Shee, provides that it shall be lawful for friendly societies to contribute to institutions "by which services are rendered to persons who are members of the society such sums as will meet the total expenditure actually incurred by these institutions or societies on behalf of such members, and any sum so expended shall be treated as expenditure on such benefits under this part of the Act." The position of pupil nurses in hospitals was explained by the Chancellor, in reply to a question asked by Sir Samuel Scott; if no wages are paid the hospital is responsible for the employee's as well as the employer's contribution, but the matter is under consideration.

